# An assessment system for clinical and biological interpretability in ulcerative colitis

**DOI:** 10.18632/aging.205564

**Published:** 2024-02-16

**Authors:** Shiqian Zhang, Ge Zhang, Wenxiu Wang, Song-Bin Guo, Pengpeng Zhang, Fuqi Wang, Quanbo Zhou, Zhaokai Zhou, Yujia Wang, Haifeng Sun, Wenming Cui, Shuaixi Yang, Weitang Yuan

**Affiliations:** 1 Department of Colorectal Surgery, The First Affiliated Hospital of Zhengzhou University, Zhengzhou 450052, Henan, China; 2 Department of Cardiology, The First Affiliated Hospital of Zhengzhou University, Zhengzhou 450052, Henan, China; 3 Henan Province Clinical Research Center for Cardiovascular Diseases, Zhengzhou 450052, Henan, China; 4 Department of Neonatology, The Third Affiliated Hospital of Zhengzhou University, Zhengzhou 450052, Henan, China; 5 Department of Medical Oncology, Sun Yat-Sen University Cancer Center, Guangzhou 510060, Guangdong, China; 6 State Key Laboratory of Oncology in South China, Collaborative Innovation Center of Cancer Medicine, Sun Yat-Sen University Cancer Center, Guangzhou 510060, Guangdong, China; 7 Department of Thoracic Surgery, The First Affiliated Hospital of Nanjing Medical University, Nanjing 210029, Jiangsu, China; 8 Department of Urology, The First Affiliated Hospital of Zhengzhou University, Zhengzhou 450052, Henan, China

**Keywords:** machine learning, diagnosis model, regulatory landscape, computational biology, biological interpretability

## Abstract

Ulcerative colitis (UC) is a serious inflammatory bowel disease (IBD) with high morbidity and mortality worldwide. As the traditional diagnostic techniques have various limitations in the practice and diagnosis of early ulcerative colitis, it is necessary to develop new diagnostic models from molecular biology to supplement the existing methods. In this study, we developed a machine learning-based synthesis to construct an artificial intelligence diagnostic model for ulcerative colitis, and the correctness of the model is verified using an external independent dataset. According to the significantly expressed genes related to the occurrence of UC in the model, an unsupervised quantitative ulcerative colitis related score (UCRScore) based on principal coordinate analysis was established. The UCRScore is not only highly generalizable across UC bulk cohorts at different stages, but also highly generalizable across single-cell datasets, with the same effect in terms of cell numbers, activation pathways and mechanisms. As an important role of screening genes in disease occurrence, based on connectivity map analysis, 5 potential targeting molecular compounds were identified, which can be used as an additional supplement to the therapeutic of UC. Overall, this study provides a potential tool for differential diagnosis and assessment of bio-pathological changes in UC at the macroscopic level, providing an opportunity to optimize the diagnosis and treatment of UC.

## INTRODUCTION

UC is a type of remitting and relapsing IBD characterized by inflammation of the colonic mucosa, which originates in the distal colon and can extend proximally to encompass the entire colon. The etiology of UC is thought to result from a complex interplay of environmental factors, the gut microbiome, the immune system, and genetic predisposition. Common clinical manifestations of UC include diarrhea with blood, frequent abdominal pain, fatigue, and fecal incontinence [1, 2]. In addition, data from various studies have demonstrated a growing incidence of UC in developing countries, particularly in Asia. Previously considered low-risk populations, such as those in Japan and India, have also witnessed an increase in UC incidence, with rates ranging from 0.5 to 31.5 cases per 100,000 individuals annually [3, 4].

Timely and accurate diagnosis and treatment of UC are very important. At present, there is no single gold standard model for the diagnosis of UC, and although endoscopy, ultrasound, histological analysis, and various biomarkers, such as fecal calcitonin and fecal milk protein, are increasingly used in noninvasive diagnosis and detection, there is no obvious advantage in the early diagnosis of the disease [5–7]. To date, genome-wide association studies (GWAS) have identified numerous UC susceptibility sites [8]. Despite these advancements, limited progress has been made in the early diagnosis and understanding of UC from a molecular genetic perspective. Hence, there is a pressing need to establish a reliable molecular genetic marker for early diagnosis and improved clinical management of the disease. Machine learning is increasingly being utilized in various fields due to its ability to be integrated with a wide range of variables. In the medical field, the maturity of this technology has been demonstrated in the diagnosis of Alzheimer’s disease and breast cancer [9, 10].

In this study, we performed a computational systems biology approach to decode the transcriptome profiles of patients with different severities of UC at the bulks and single-cell levels. Using a machine learning program integrating eight learners, we identified ulcerative colitis relative biomarker signatures to build a robust diagnostic model. Although our diagnostic model has high performance in distinguishing between normal people and UC patients, one of the limitations of machine learning is the lack of inherent biological interpretability of the model. Therefore, we built an unsupervised quantitative scoring system called UCRScore to quantify disease status and biological mechanisms through the expression profile of UC-related genes [11]. We also performed single-cell RNA sequencing in mice to understand the underlying molecular mechanisms involved in UCRScore in UC states. Overall, our findings provide a novel predictive characterization and quantification system to evaluate diagnostic models and underlying mechanisms of UC occurrence.

## MATERIALS AND METHODS

### Data acquisition and collection

In this study, a total of 606 human samples were obtained from 10 datasets obtained from the Gene Expression Omnibus (GEO) database. All data were processed by de-batch effect and normalization, including meta-cohort GSE87466 for model construction (21 Healthy controls, 87 UC patients), five independent external model-validated datasets including GSE47908(19 Healthy controls, 41 UC patients), GSE59071(16 Healthy controls, 92 UC patients), GSE75214(19 Healthy controls, 100 UC patients), GSE92415(20 Healthy controls, 54 UC patients) and GSE14580(6 Healthy controls, 24 UC patients). There are also four UC cohorts at different stages to validate the unsupervised quantitative scoring system we constructed, including GSE53306 (16 Healthy controls, 12 Inactive UC, 12 Active UC), GSE16879 (8 Infliximab Responsive UC, 16 Infliximab Irresponsive UC), GSE13367(16 Infiltrative UC, 18 Non-infiltrative UC), GSE6731(5 Infiltrative UC, 4 Non-infiltrative UC). All downloaded dataset details are shown in Supplementary Table 1.

### Omics data resource

C1-C8 and Hallmark biological datasets were retrieved from the Molecular Signatures Database (MSigDB) (http://www.gsea-msigdb.org/gsea/msigdb). The list of TFs was derived from the Cistrome database (http://cistrome.org/). Human regulated signaling pathways were extracted from the Biocarta database (https://maayanlab.cloud/Harmonizome/dataset/Biocarta+Pathways). Human protein and biological interaction signatures were derived from Reactome Pathway Database (https://reactome.org). Drug signatures and gene expression profiles were downloaded from Connectivity Map (CMap) database (http://www.broadinstitute.org).

### Differential expression analysis and construction of weighted correlation network analysis (WGCNA)

The GSE87466 dataset was utilized as a meta-cohort and the gene expressions were matched according to the GPL13158 platform. After averaging the expression values of equivalent genes and eliminating missing values, a total of 20277 filtered genes were obtained. Differential expression analysis was performed with the threshold for DEGs set at P = 0.05 and | log2 Fold Change (FC) | >1. Differential gene expression analysis was conducted using the Limma and sva packages according to previous studies to identify Differentially Expressed Genes (DEGs) [12, 13]. WGCNA is used to screen dysregulated gene co-expression patterns that promote UC occurrence. The levels of gene expression were systematically organized in descending order according to their standard deviation. Subsequently, the 5000 genes exhibiting the highest variation were selected for further analysis. To ensure analytical validity, hierarchical clustering analysis was performed, wherein outlier samples were methodically excluded. Additionally, the Pearson correlation coefficient was calculated for each pair of genes, facilitating the construction of a comprehensive gene similarity matrix. The adjacency matrix was converted into a topological overlap matrix (TOM) and its complementary form, 1-TOM, to effectively reflect gene similarities and dissimilarities, respectively. Subsequently, genes were stratified into distinct modules via hierarchical clustering. We then computed the module eigengene (ME) for each module to represent its respective profile. Notably, modules showing a high correlation with the occurrence of UC were pinpointed, indicating our key dysregulated gene co-expression patterns. The parameter settings employed in our analysis were as follows: a soft thresholding power β = 14, a minimum module size = 50, a merge cut height = 0.25, and a deepSplit value of 2 [14].

### Calculation of the global divergence between a pair of expression profiles

The global divergence between a pair of gene expression profiles was calculated as Euclidean distance:


$$\text{RMSD}=\sqrt{\sum_{i=1}^{n}{\left(log2xi-log2yi\right)}^{2}\text{/}n}$$

where *xi* and *yi* are the expression of gene *i* in expression profiles, respectively, and *n* is the number of genes present in the expression profile.

### UCRGs generated from interactive learning

A comprehensive program incorporating eight distinct machine-learning algorithms was developed to identify a signature of occurrence UC. This ensemble included: Backpropagation neural network (nnet), random forest (rf), boosted generalized linear model (glmboost), lasso and elastic-net-regularized generalized linear models (glmnet), bootstrap aggregation classification and regression Trees (Bagged CART), generalized liner model (glm), partial least squares (pls), and classification and regression trees (CART).

The UCRG signature generation procedure was as follows:

1. The common relative DEGs obtained were utilized to construct a diagnostic model for occurrence of UC.
2. The initial exploration of UC relative gene signature (UCRGs) was conducted in meta-cohort GSE87466, which was randomly divided into training and testing cohorts in a 7:3 ratio.
3. The eight learners were performed on common relative DEGs to fit models separately. To prevent overfitting caused by the complex model, 10-fold 100-repeated cross-validation (cv) was adopted to improve the generalization ability of the training cohort.
4. Based on a consensus assessment strategy, the effectiveness and applicability of all models were assessed in the testing cohort, including Recall, Precision, F1, Accuracy, Harrell’s Concordance Index (C-index) and Root Mean Square of Residual (RMSR).
5. For feature selection, the UCRGs was created utilizing the random forest-Recursive Feature Elimination (RF-RFE) strategy combined with a 10-fold 10-repeated cv and the decreasing accuracy method (Gini coefficient method). The average model error rate of the input genes was calculated, and the optimal number of trees in the random forest model was set at 250.

### Construction of diagnostic model based on UCRGs classifier

The diagnostic model of UCRGs was performed through the use of a neural network model constructed with the neuralnet package [15]. The model parameters were set to include two hidden layers of 10 and 6 nodes, which were used to obtain gene weight information to construct a diagnostic model. The expression data of the UCRGs were first transformed into “Feature Score” based on their expression level (above or below the median). In the case of a certain sample, the expression value of a specific gene was compared to the median of all sample expression values of that sample. If the expression value of the upregulated gene is higher, it will be valued as 1, otherwise 0. Similarly, if the expression value of downregulated gene is higher, it will be valued as 0, otherwise 1 (Supplementary Table 4). Ultimately, we utilized the “Feature Score” sheet, comprising rows representing samples and columns indicating selected features along with the outcome variable, for training the backpropagation neural network, thereby constructing the diagnostic model classifier [9].

**Table:** 

**Feature score matrix**	**Expression level**	
	**Low**	**High**
Upregulated	0	1
Downregulated	1	0

### Verification of external independent dataset

The diagnostic model based on UCRGs was validated on five independent external datasets including GSE47908, GSE59071, GSE75214, GSE92415 and GSE14580. The classifier’s generalizability across independent external cohorts was ascertained through a suite of performance metrics: sensitivity, specificity, accuracy, Kappa, precision, recall and F1-score.

### Establishment of UCRScore

One of the most difficult aspects of using the machine learning algorithm is its lack of interpretability. To overcome this issue, we used an unsupervised approach that allows us to acquire insights regarding a person’s disease state from both a biological and clinical standpoint. The identified UCRGs were subjected to Principal Coordinates Analysis (PCoA). PCoA is a model of multidimensional scaling that originally simplifies the data sets by projecting it in a space spanned by orthogonal axes that are few in numbers [16].

1. Based on UCRGs, we carried out an analysis of similarities (ANOSIM) and PCoA. The R package Vegan’s vegdist function was used to determine Bray-Curtis diversity, whereas beta_diversity.py was used to create the Bray-Curtis dissimilarity matrix.
2. The PERMANOVA test (2-way adonis) was used to determine if significant differences existed between groups.
3. We developed a quantification system UCRScore, designed to assess the vulnerability of ulcerative colitis and the severity of the disease [11]:
$$\text{UCRScore}=(PCoA1\;score+PCoA2\;score\times \sum exp_{i}$$

where *exp_i_* is the expression level of occurrence UC-related genes. *PCoA1 score* and *PCoA2 score* are the first two principal components produced in PCoA.

### Cellular heterogeneity underlying UCRScore

For the quantification of various cell types’ proportions within the UC microenvironment, we utilized xCell, a gene signature-based tool for cell type enrichment. xCell computes cell abundances by analyzing millions of transcripts from 64 immune and stromal cell types, employing data derived from an extensive array of pure cell types. This technique effectively delineates closely related cell populations, ensuring an accurate representation of cellular heterogeneity.

### Construction of the UCRGs global regulatory landscape

We constructed a multidimensional regulatory network to uncover potential regulation mechanisms underlying UCRGs.

1. We first identified multi-dimensional components dysregulated significantly between normal and UC. This involved retaining upstream dysregulated transcription factors (TFs) with absolute logFC>0.5 and FDR<0.01, dysregulated hallmark signatures (Hallmarks) with absolute t>2 and FDR<0.01, dysregulated pathways signatures (Pathways) with absolute t>5 and FDR<0.01, dysregulated biochemical reactions (Reactome) with absolute t>5 and FDR<0.01, and differences in cell compositions with absolute t>2 and FDR<0.01.
2. Pearson and Spearman correlation analyses were applied respectively to normally and non-normally distributed data for co-analyzing the interaction coefficients among upstream UCRGs panel, TFs, Hallmarks, Cells, as well as downstream Pathways and biochemical reactions. Components exhibiting a |correlation coefficient| < 0.5 and a P-value <0.001 were rigorously selected based on their prominent spatial correlation with UCRGs. Subsequently, regulatory networks were delineated utilizing the igraph package.

### Computational novel therapy discovery and repurposing

The Connectivity Map (CMap) database containing multiple drug-specific genomic expression profiles was used to explore potential compounds for UCRGs in this study:

1. The eXtreme-Sum signature matching methodology was employed to align identified patterns with pharmacological perturbation data [17]. This technique involves querying pharmacological perturbation datasets to identify compounds capable of reversing these identified patterns. It calculates the cumulative changes in pharmacological signatures corresponding to the upregulated (sumup) and downregulated (sumdown) disease genes. The XSum score is subsequently calculated as the sum of these values:
XSum score=sumup+sumdown
2. The randomization method was employed to ascertain the statistical significance of the findings. Drug compounds exhibiting a predicted XSum score beneath the established threshold were inversely ranked according to their scores. Notably, compounds demonstrating considerably negative scores are characterized by gene expression patterns inversely related or contrary to those associated with the initiation and occurrence of UC, thus suggesting potential new therapeutic applications.

### Dynamic changes analysis and dynamic network biomarker analysis

Dynamic network biomarkers (DNB) serve as universal early warning system to provide insight into impending critical transitions before branching or sudden deterioration occurs disease evolution begins within a ‘normal state’ a phase predominantly stable yet subject to incremental modifications. Preceding the onset of pathological conditions, the ‘pre-disease state’ acts as a critical juncture, denoting an imminent and marked shift towards pathology. This transition culminates in the ‘disease state’, a fundamentally altered, stable condition, often signifying an irreversible straying from normalcy. In accordance with the DNB theory, a collection of molecules exists in the pre-disease state that holds potential for disease prediction. We then used the common DEGs expression profiles to identify DNB according to the nonlinear dynamic theory.

1. A marked escalation in the mean SD of DNB molecular components.
2. A significant elevation in the mean PCC observed amongst DNB molecules during the pre-disease phase.
3. A considerable reduction in the average PCC between DNB molecules and non-DNB entities within the identical state.

These three criteria can be quantified as a module Composite Index (CI), where SD represents the average standard deviation (SD) of molecules within the DNB module, PCC denotes the average absolute Pearson correlation coefficient (PCC) among molecules within the module, and PCC signifies the average absolute PCC between DNB molecules and non-DNB molecules. It is anticipated that the CI undergoes a sudden and significant increase just prior to the critical transition to the disease state, serving as a robust early warning signal.

### Single-cell characteristics analysis

We downloaded the GSE182272 single-cell sequencing set from GEO database, to verify the cellular and molecular mechanism of UCRScore analysis at the single-cell level, three selected UC mice samples were subjected to analysis. Single-cell gene expression data were processed using the Seurat pipeline. Integration of cells across different samples was achieved through the “FindIntegrationAnchors” and “IntegrateData” functions. The top 2000 variable genes, identified via “vst” selection, served as input features for PCA-based dimensionality reduction. Subsequently, the first 20 principal components (PCs) deemed significant by jackstraw analysis were utilized in UMAP for enhanced reduction in dimensionality and visualization of clustering [18, 19]. For each cell, UCRGs underwent AUCell scoring, utilizing the area under the curve (AUC) to rank gene expression and gauge the proportion of highly expressed gene sets. The “AUCell_exploreThresholds” function was applied to establish thresholds for identifying cells actively featuring UCRGs. Discrimination analysis and quantification, conducted via the Cell cycle scoring function, utilized predefined genes associated with the cell cycle. This analysis facilitated the projection of cells onto UMAP space, with subsequent coloring based on cell cycle clustering, enhancing visualization. Intercellular communication networks were analyzed from single-stranded RNA-seq data with the CellChat package [20]. In the single-cell RNA-seq dataset, the UCRScore was defined as the average expression level of UCRGs per single cell. The estimation of UCRScore is achieved by using the “AUCell” package [21].

### Statistical analysis

All statistical analyses were carried out using R 4.1.2 software (https://www.r-project.org). Pearson correlation analysis was used to explore the correlation between variables, t-tests were used to compare normally distributed continuous variables between two groups, for comparisons of more than two groups, the Kruskal-Wallis test was used to compare differences. The mean ± standard deviation for descriptive statistics was used for continuous variables with a normal distribution, and the chi-squared test was applied to compare categorical variables. *P*-values were two-sided and lower than 0.05 was considered statistically significant.

### Availability of data and materials

The data sets analyzed in this study are available in the GEO database (https://www.ncbi.nlm.nih.gov/geo/). All of the multiple microarrays are derived from this database. The original contributions presented in this study are included in the materials and supplementary materials.

## RESULTS

### Revealing the dysregulated pattern of gene co-expression of UC occurrence

**Figure 1:**
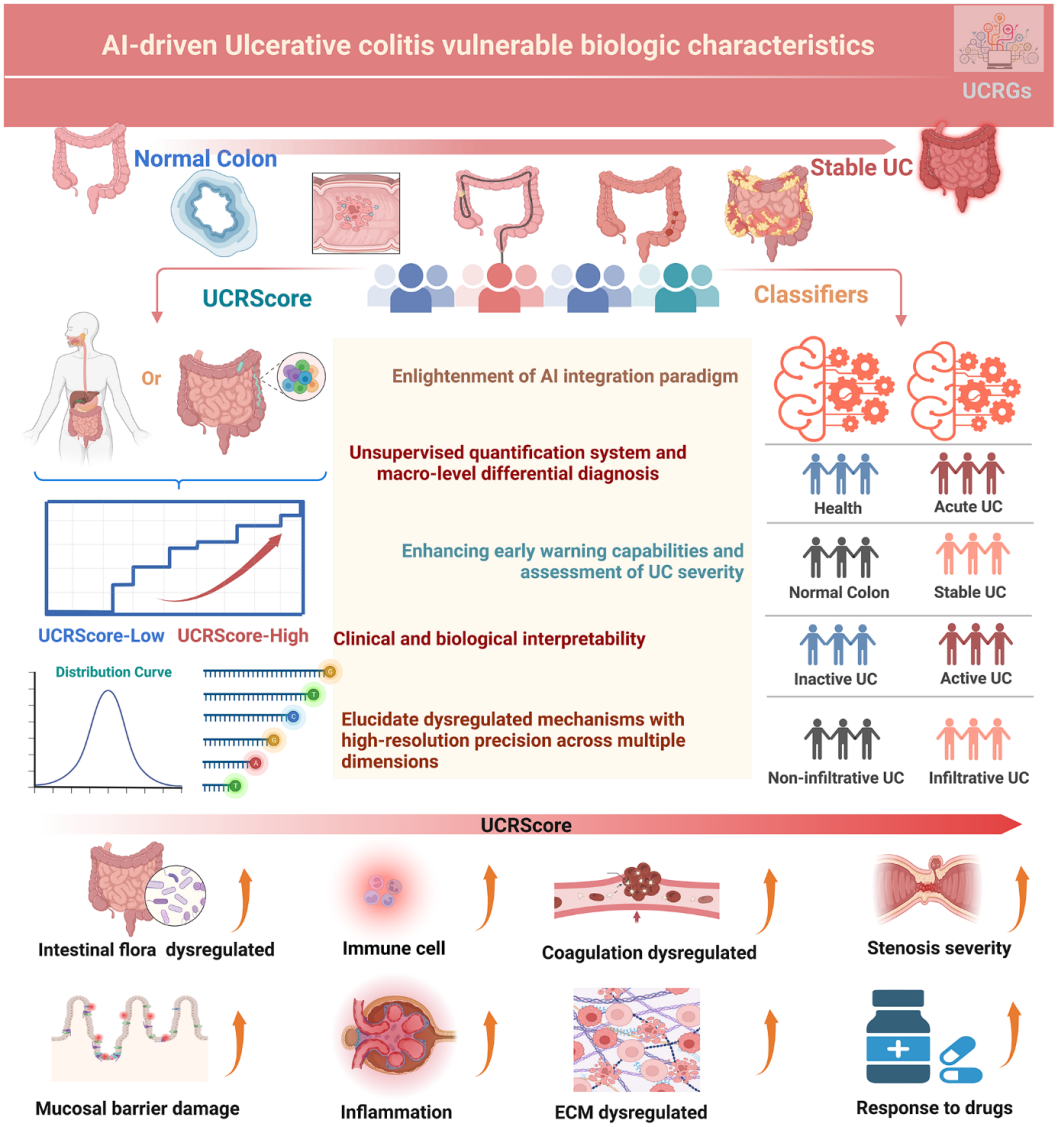
Graphical abstract of this study.

**Figure 2:**
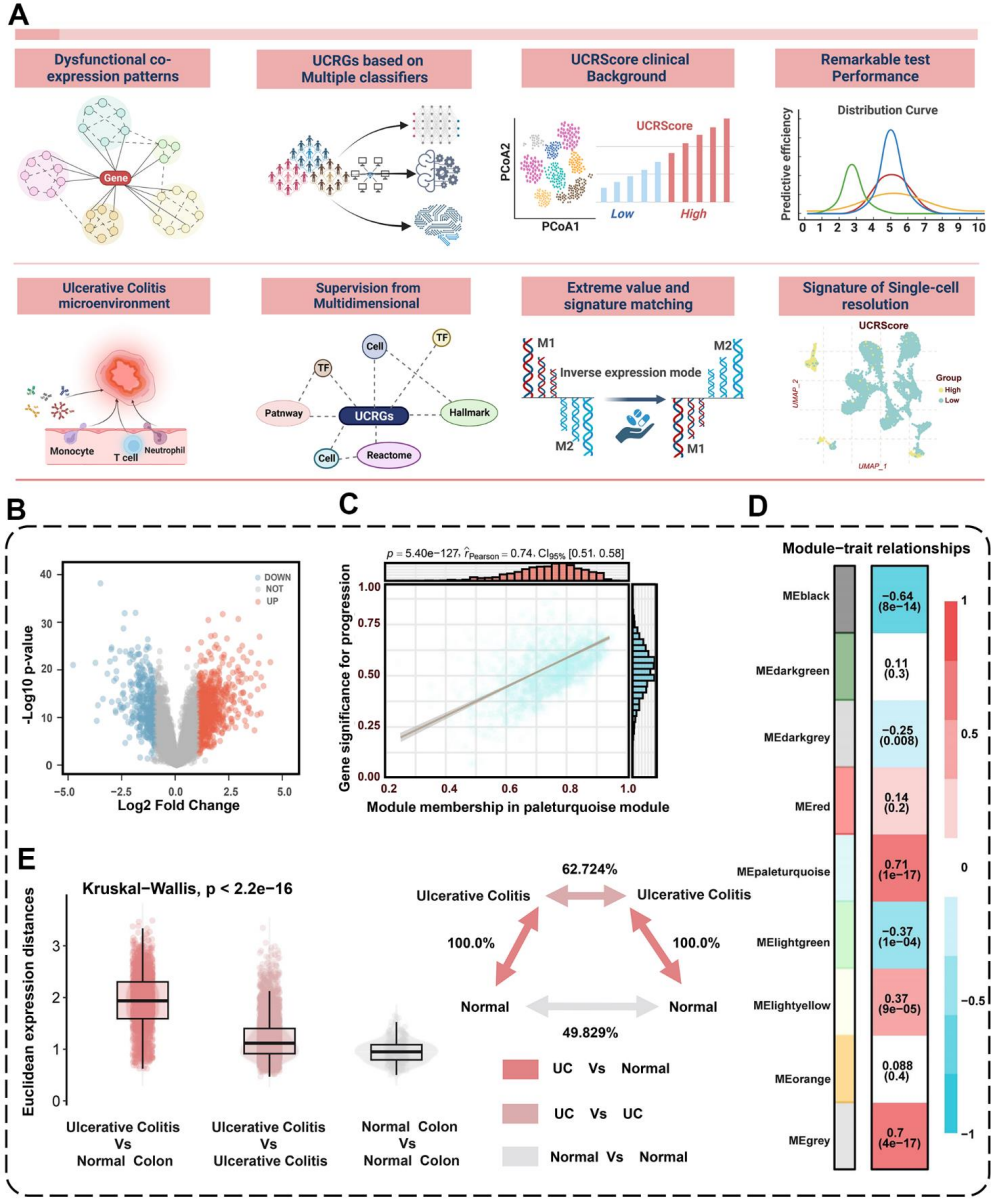
**Identification of dysregulated co-expression modules.** (**A**) Flowchart of the research design. (**B**) Volcano plot of differentially expressed genes between normal and UC patients. The volcano plot displays the log2 fold change on the x-axis and -log10(p-value) on the y-axis. (**C**) The significant associations among gene significances, module memberships, and the disease severity in the turquoise module. (**D**) Correlation between module eigengenes and the occurrence of UC. (**E**) Global differences in differential gene expression between UC patients and normal people in the GSE87466 cohort. The Euclidean expression distances were calculated between UC and normal (red), within samples of UC (lilac), and within samples of normal (grey). The inset summarizes the average distances between pairs of tissues as a percentage of the average distance between UC and normal people.

A graphical abstract of the study is shown in Figure 1. The main flowchart of the analysis is depicted in Figure 2A. The results showed 866 up-regulated and 404 down-regulated DEGs based on the cohort GSE87466 (Figure 2B). WGCNA was then conducted with a soft threshold β set to 14 (no scale, R^2^ = 0.859), providing an appropriate power value for network construction (Supplementary Figure 1A, 1B). The minimum size for the gene dendrogram was set to 30 (Supplementary Figure 2A), Subsequently, the adjacency matrix was transformed into a TOM to facilitate more straightforward module segmentation, as illustrated in Supplementary Figure 2B. This transformation enabled the identification of 9 co-expression modules via hierarchical clustering, detailed in Figure 2D. The module most significantly associated with the disease was determined by Pearson correlation analysis. The eigengene (first principal component of gene expression within a module) was considered as the representative of the module (Figure 2C). The highest correlation in the module-trait relationship was observed between the turquoise module (Figure 2D). In the turquoise module, the correlation coefficient between gene significance (GS) and module membership (MM) reached 0.74, which suggested that the quality of module relevant to UC construction was superior. To seek the hub genes from the top relevant turquoise modules, we filtered out those not significantly dysregulated between normal and UC patients based on the cohort GSE87466, and identified 746 common DEGs as the dysregulated co-expression pattern genes (Supplementary Figure 3A). We subsequently conducted an analysis focused on UC and employed the Euclidean distance metric as a measure of divergence between pairs of expression profiles [22]. This allowed us to delve deeper into the comprehensive changes in relative gene expression among UC and compare them to the normal tissues within the meta-cohort. Notably, we observed that the expression divergence between UC and their corresponding normal tissues, as well as within UC samples, was significantly higher than the divergence found within normal samples (Figure 2E). Furthermore, we conducted an over-representation analysis (ORA) of common DEGs using gene sets obtained from the MSigDB of C1 to C8 and Hallmark gene sets [23]. The results unveiled a substantial enrichment of gene sets associated with inflammatory responses, cellular activation and T-cell activation, as illustrated in Supplementary Figure 3B.

### Machine learning-based diagnostic models and generated UCRGs

**Figure 3:**
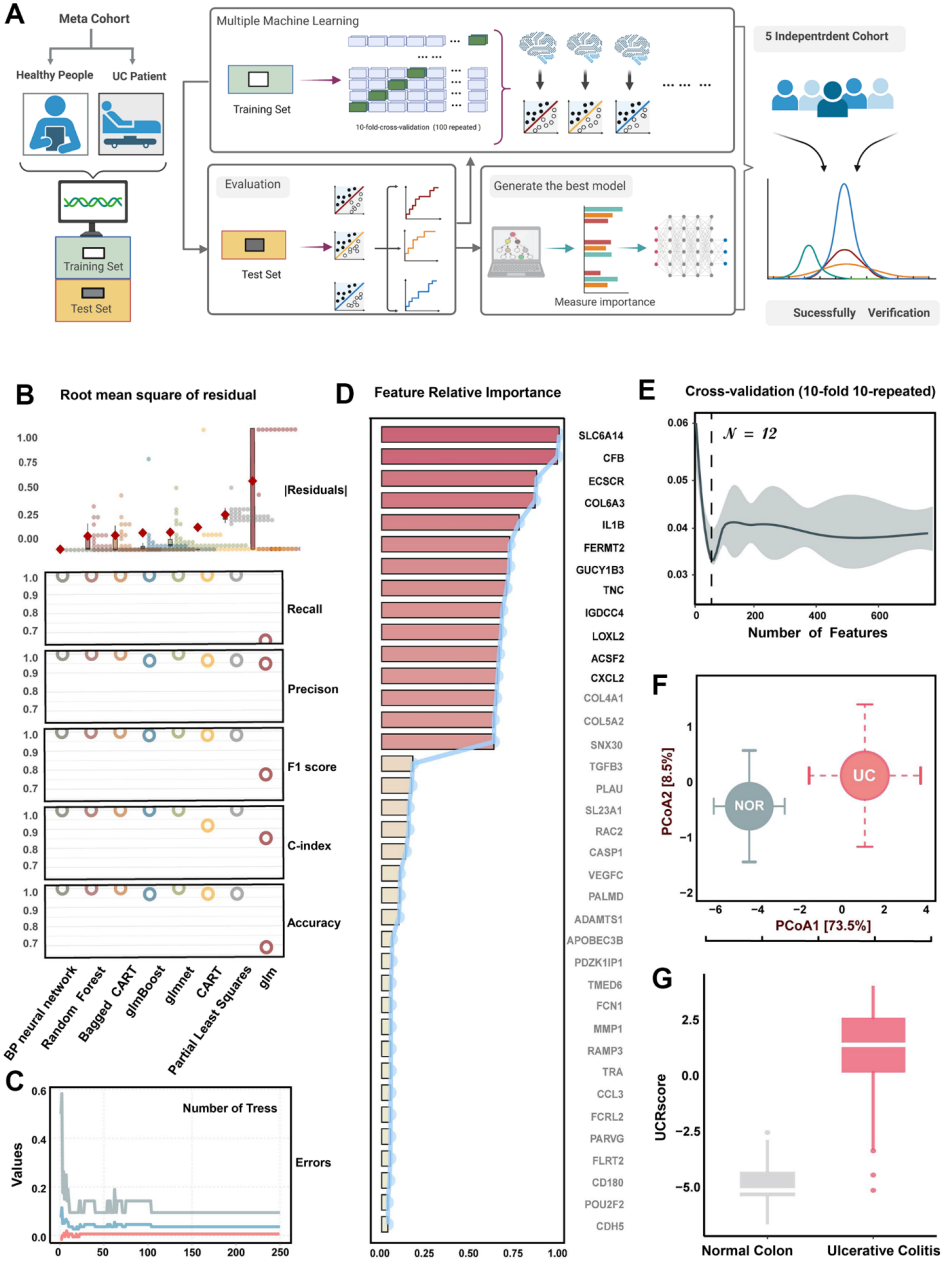
**Machine learning-based integrative program generates UCRGs.** (**A**) A schematic overview of the machine learning process based on the UCRGs development integration model. (**B**) Comprehensive performances of eight types of learners. Box plots depicted the distribution of the residuals, with red highlighted dots representing root mean square of residuals (RMSR). Circles showed the distribution of recall, precision, F1-score, C-index, and accuracy of each learner. (**C**) The influence of the number of decision trees on the error rate. The x-axis represents the number of decision trees, and the y-axis shows the error rate. (**D**) The importance of common DEGs varies. The barplot shows the distribution of the average decreasing Gini coefficients, while the line chart shows the average decreasing accuracy. The top 12 genes were identified as UCRGs. (**E**) Using 10-fold 10 repeated cross-validation combined with decreasing precision method (based on Gini coefficient of random forest) to eliminate the recursive features of commonDEGs, reduce the dimension of feature space and avoid over-fitting. When the number of variables is set to 14, the error is minimized. (**F**) Principal coordinate analysis of Bray-Curtis dissimilarities obtained for the UCRGs expression profiles in the GSE87466 cohorts. The circles and error bars indicated the mean and standard errors of the mean. (**G**) The distinction of UCRScore in the groups of meta-cohort.

The flow chart of screening UC relative genes by machine learning is presented in Figure 3A. In order to fit models, we used eight classical learners and performed 10-fold cross-validation with 100 iterations. In the testing cohort, we assessed each learner’s performance using five metrics: accuracy, C-index, F1-score, precision, recall, and RMSR. Based on these performance criteria, the random forest and back propagation neural network displayed higher classification abilities (Figure 3B). The common DEGs were input into the random forest classifier, and the minimum fault tolerance rate stabilized when the tree reached 250 (Figure 3C). Next, we performed the recursive feature elimination cross-validation integrated with the random forest-based Gini coefficient method, and 12 genes with the highest importance were selected as UCRG panel (Figure 3D, 3E) [24]. The top 12 genes, including SLC6A14, CFB, ECSCR, COL6A3, IL1B, FERMT2, GUCY1B3, TNC, IGDCC4, LOXL2, ACSF2, and CXCL2, were selected as UCRGs panel for further analysis, which suggested that these genes may play an important role in the occurrence of UC. The location of these UCRGs panel on chromosomes, as well as their co-expression and shared protein domains with tissue sample genes, were also identified (Supplementary Figure 4A, 4B). Principal Coordinate Analysis (PCoA) of UCRGs panel expression profiles in the GSE87466 cohort revealed significant differences in the characterization of UCRGs between UC patients and normal people. (p < 0.001, 1000 permutations of the PERMANOVA test, Figure 3F).

### Robust performance of UCRGs-based diagnostic model

**Figure 4:**
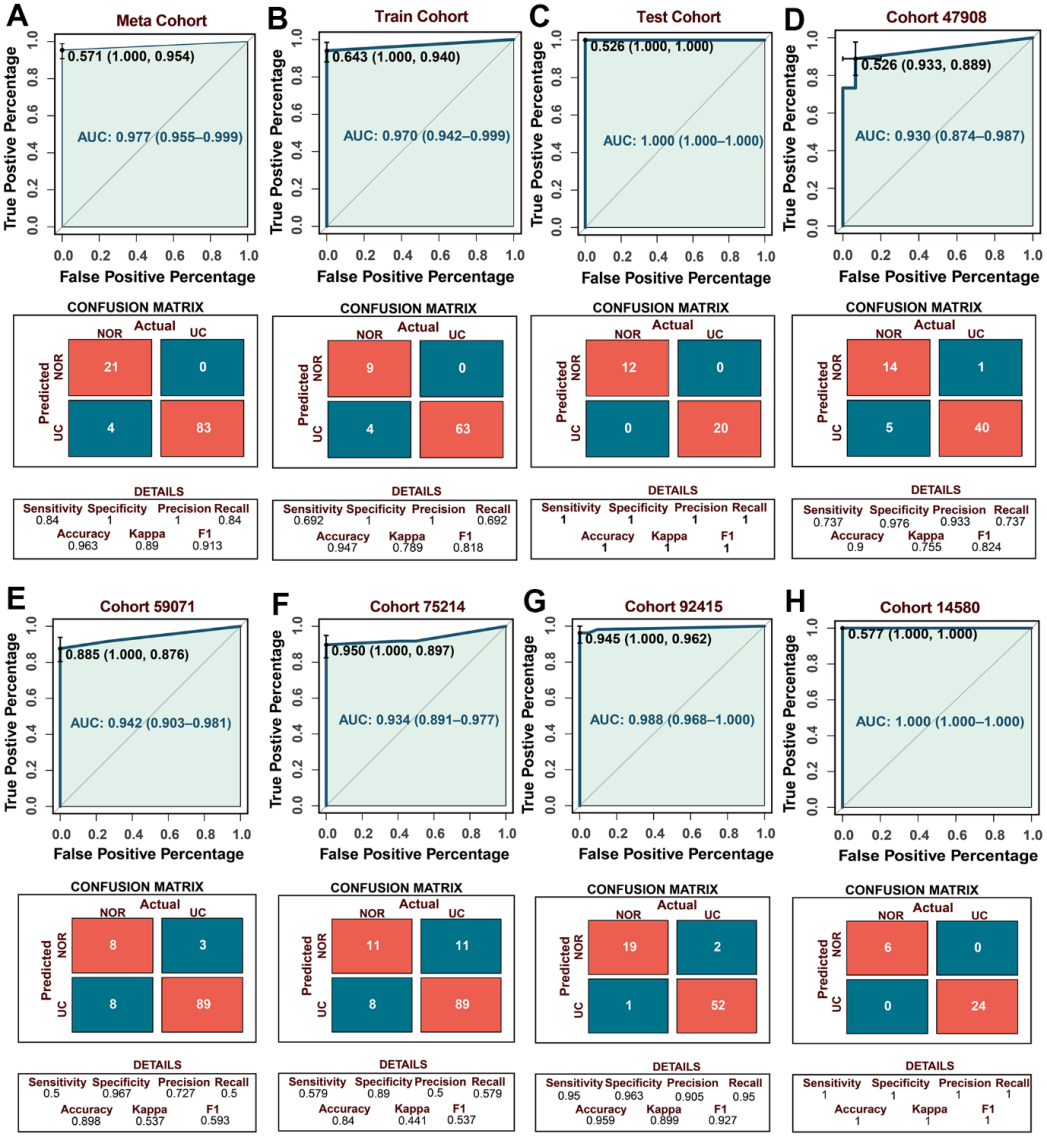
**Establishment of the UC diagnostic model and verification of external independent cohort.** (**A**–**C**) The predictive performance of the diagnostic model in the meta, training, and testing cohort. (**D**–**H**) The predictive performance of the diagnostic model in the external independent cohort. The confusion matrices showing the detailed results of discriminant analyses by UCRGs classifier model in five independent cohorts.

Using back propagation neural network classifier from UCRGs to construct patient diagnosis model (Supplementary Table 3). To verify the accuracy and universality of the diagnostic model, receiver-operator characteristic (ROC) analysis was used to preliminarily measure the discrimination of the model, the area under the ROC curve (AUC) [95% confidence interval] of the diagnostic model in the meta-cohort GSE87466, training set and testing set was 0.977 [0.955-0.999], 0.970 [0.642-0.999], and 1.000 [1.000-1.000], respectively (Figure 4A–4C). To further verify the accuracy of neural network diagnostic model from the outside, five external independent datasets were selected to verify the model, with AUCs of 0.930 [0.874-0.987] in GSE47908; 0.942 [0.930-0.987] in GSE59071; 0.934 [0.891-0.977] in GSE75214; 0.988 [0.968-1.000] in GSE92415; 1.000 [1.000-1.000] in GSE14580, all the AUC values show convincing diagnostic performance (Figure 4D–4H). The confusion matrix provides detailed discriminant analysis results. The higher precision, specificity, kappa, F1 score, accuracy and AUC consistently indicate that the back propagation neural network classifier based on UCRGs can stably distinguish normal people from UC patients, the diagnostic model has high accuracy and excellent performance.

### The biological implication and immune landscape underlying UCRScore

**Figure 5:**
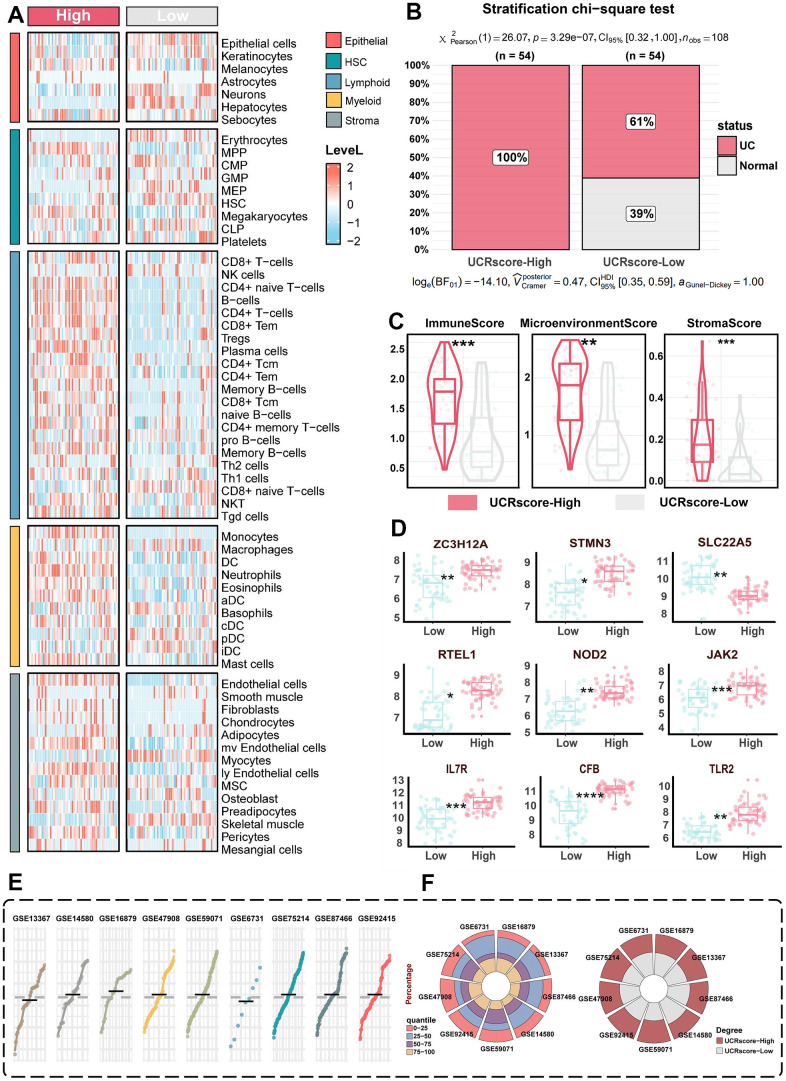
**The biological significance of UCRGs.** (**A**) Heatmap shows the abundance distribution of five cell types in different UCRScore groups. (**B**) Chi-square test shows that the proportion of different scores in the two groups. (**C**) The distribution of xcell quantification between UCRScore-High and UCRScore-Low groups comparison of differences between high and low UCRScore in xCell score. (**D**) Distribution of genes related to UC occurrence between high and low UCRScore groups. (**E**) UCRScore was calculated among UC individuals in nine bulk cohorts. The gray dashed line represents the median UCRScore of all samples. (**F**) Left: Barplot t showing proportion of UCRScore quartiles across nine bulk cohort. Right: Percentage of samples classified as UCRScore-High and UCRScore-Low in nine bulk cohorts, with the median UCRScore as the cutoff (gray dashed line in the above gene panle).

To overcome the interpretability challenges associated with machine learning classifiers, we devised a novel unsupervised scoring system, designated UCRScore, which measures the vulnerability and severity of UC prevalence at both overall and single-cell resolution, and divides it into high and low groups based on the median scores (Figure 3G). The chi-square test further validated the difference in scores between the different groups, with higher scores accounting for a greater proportion of patients with UC (Figure 5B). Subsequently, the relationship between UCRScore and histiocyte characteristics was analyzed. The cellular heterogeneity landscape in the UC microenvironment was quantified, and it was found that most immune cells, such as neutrophils, T cells, and B cells, as well as stromal cells, such as fibroblasts and endothelial cells, exhibited higher expression in the high score group (Figure 5A). The xcell algorithm quantification highlighted an overstimulated immune activity, microenvironment perturbation, and stroma density in the UCRScore-High lesions (Figure 5C). In addition, we collected previously reported signature genes associated with the occurrence and development of UC (Supplementary Table 2) and compared the score with their predictive effects in meta-cohort. The results showed that almost all of the high score groups had an elevated risk of developing the disease, and thus the accuracy of our system for the other variables improved (Figure 5D). More than 500 UC relative people were involved in 9 bulk cohorts (GSE87466, GSE47908, GSE59071, GSE75214, GSE92415, GSE14580, GSE16879, GSE53306 and GSE6731) based on their UCRScore median, we were able to classify these people into UCRScore-High and UCRScore-Low subtypes. The distribution pattern of UCRScore is similar, and the proportions of the two groups are comparable, indicating that UCRScore has stable universality and generalizability (Figure 5E, 5F).

### Construction of global regulatory landscape and therapeutic discovery

**Figure 6:**
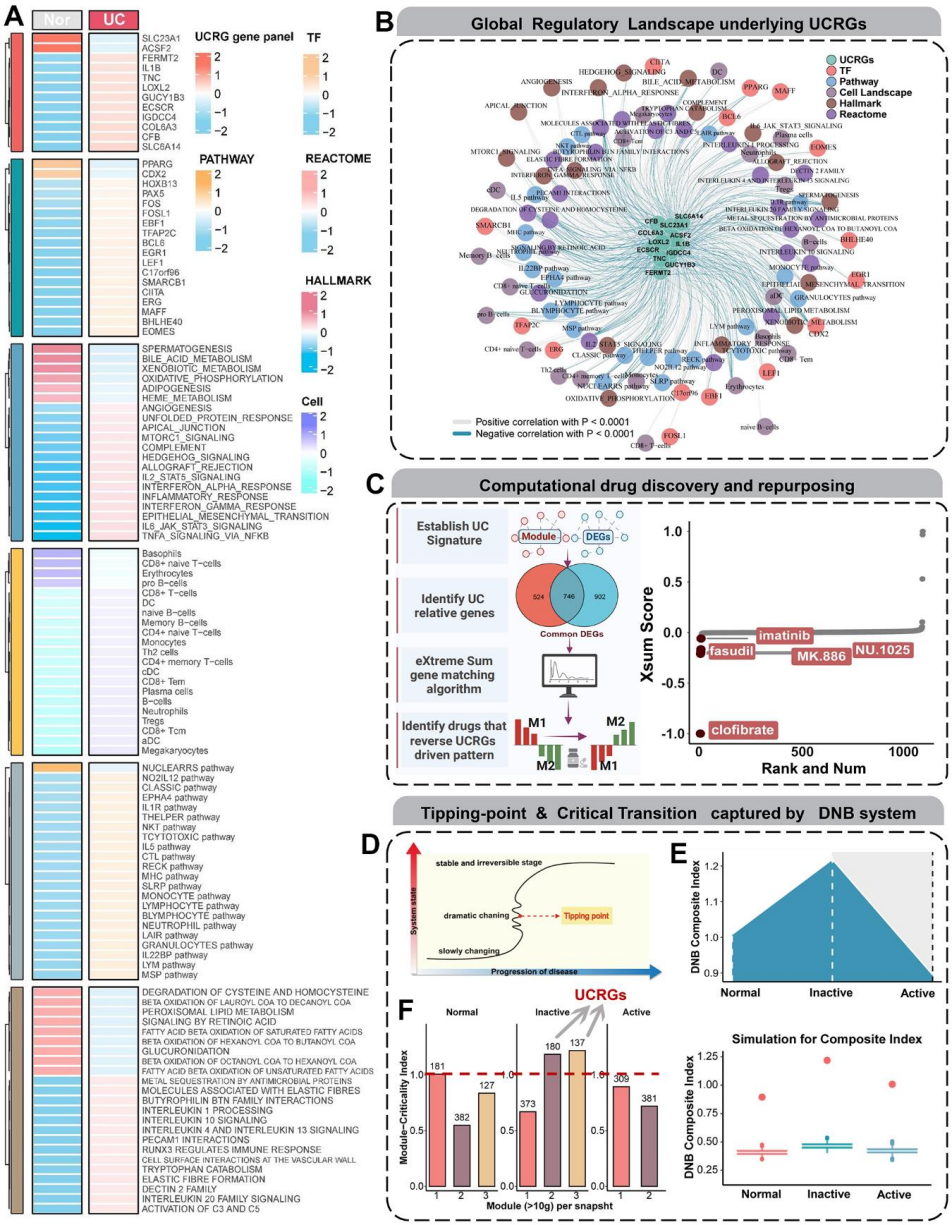
**Global regulatory landscape, benefits of therapeutic drugs and DNB analysis.** (**A**) Heatmap showing the distribution of dysregulated regulatory components from multiple dimensions. (**B**) The interaction network centered on UCRGs shows the tight modulation relationship of important regulators. (**C**) Schematic showing UCRGs-driven therapeutic discovery and results from the eXtreme Sum signature matching method. Lower scores imply higher reversal effectiveness and greater application potential. (**D**) A schematic diagram illustrates a stage transition during UC occurrence. The critical period after the early period changes the state of the biological system qualitatively and thus plays a key role in biological processes. (**E**) Line and box plots used to visualize the simulated DNBscore. The plot shows that, based on the CIs at all time points in the gene expression profile, the key transition occurs at the inactive stage. (**F**) The MCI of GSE53306 cohort validates that UCRGs exist in the inactive stage module of UC transformation.

To elucidate the intricate mechanisms contributing to UCRGs-related disease occurrence, we conducted a multidimensional analysis of dysregulated elements and synthesized them within an expansive interaction framework (Figure 6A). Utilizing a stringent threshold of an absolute correlation coefficient > 0.5 and a *P*-value < 0.001, we constructed a comprehensive regulatory network via co-expression trend analysis (Figure 6B). This network illuminated the complex interplay between key regulators, including central UCRGs panel, upstream transcription factors, downstream pathways, signaling motifs, biochemical reaction patterns, and cell infiltrates. The resultant co-expression map effectively illustrates the multifaceted regulatory interactions at play. In addition, in order to further understand the biological explanation and pathway of UCRGs, we carried out gene cluster enrichment analysis (GSEA) phenotypic analysis. Coincidentally, it also showed high biological activity in terms of neutrophil immune activity, acute inflammation and secretory membrane granules (Supplementary Figure 5). To devise targeted interventions for UC occurrence or development events, we adopted a ‘signature reversion’ strategy to counteract aberrant expression profiles (Figure 6C). Initially, we identified two distinct expression patterns that were either positively or negatively correlated with UCRScore. Subsequently, utilizing the eXtreme-Sum method, we aligned these expression profiles with pharmacological perturbation data. Notably, compounds such as clofibrate, fasudil, MK.866, NU.1025 and imatinib showed pronounced reversal effects, suggesting their viability as supplementary therapeutics to conventional treatments (Figure 6C).

### Dynamic changes analysis and DNB analysis

Subsequently, we conducted DNB analysis on datasets GSE53306, which span UC three different time points. DNB members are generally irreversible dynamic biomarkers, and this approach allowed us to identify genes closely associated with the process of UC occurrence (Figure 6D). DNBs have the potential to detect early warning signals before disease onset, thereby promoting early diagnosis [25, 26]. Therefore, our utilization of the DNB model involves measuring molecular fluctuations and correlations, with the average DNB scores for each group shown in Figure 6E. The average DNB score in the Inactive group was higher than that in the Normal and Active groups. The distribution of criticality index (CI) values indicated a strong critical state signal during the second phase, suggesting that the key transition point of ulcerative colitis lies within the Inactive stage. Subsequently, we integrated insights from the previous modules and calculated the Module Criticality Index (MCI) for commonDEGs at three different time points in the GSE53306, visualizing the results in a bar chart (Figure 6F). It is noteworthy that the MCI score was highest in the Inactive Stage, while it was lowest in the Normal Stage. Furthermore, our UCRGs were precisely situated within the modules of the critical transition stage of UC, providing further validation for our selection.

### Elucidating the biological significance of UCRScore through single-cell resolution

**Figure 7:**
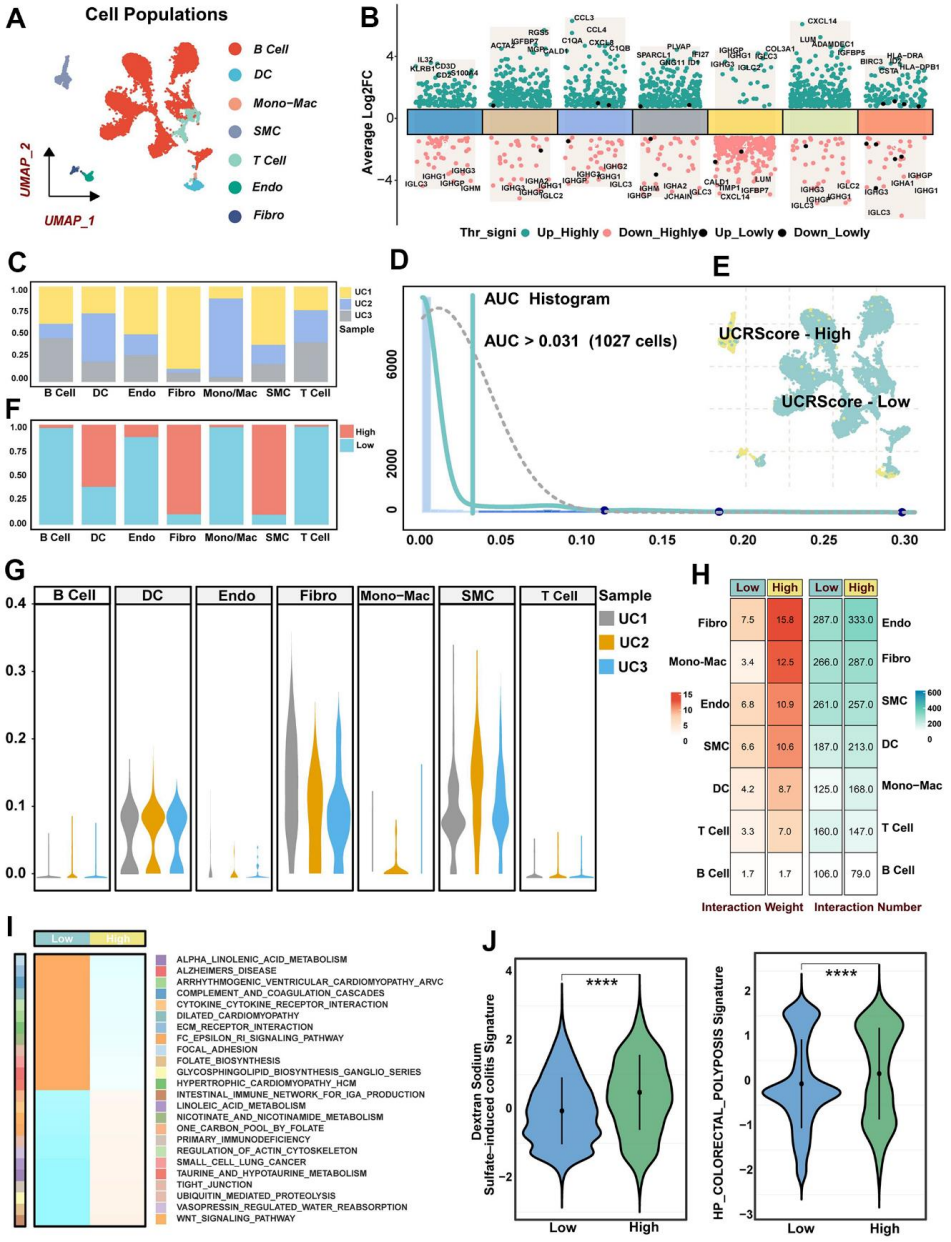
**Single-cell resolution interpretation of the biological significance of UCRScore.** (**A**) UMAP visualization showing the composition of 7 main subtypes derived from UC patient samples. (**B**) Differential expression analysis showing dysregulated genes across each cluster, with green representing up-regulated genes and red representing down-regulated genes. (**C**) The proportion of different samples in each cell cluster. (**D**) AUC area under the calculation of the UCRGs gene set in cell clusters. 1027 cells displayed higher UCRScore at a 0.031 threshold. (**E**) Cells of UC samples were colored by UCRScore. (**F**) The bar chart shows the percentage of each cell type in the two states of UCRScore-high and UCRScore-low. (**G**) The proportion of different cell cluster samples in each UC samples. (**H**) Cell-cell ligand-receptor network analysis. The number and weight interaction in different cells. (**I**) GSEA integrative analysis revealed significantly dysregulated pathways. (**J**) Distribution of published biological signatures in the UC between UCRScore-High and UCRScore-Low states.

To elucidate the mechanisms by which UCRG-induced microenvironmental alterations affect UC vulnerability, we collected disease samples containing three single-cell RNA sequences comprising 10101 cells. The filtered cells were gathered and annotated as 7 major clusters, including B cells, endothelial cells (Endo), mononuclear macrophages (Mono/Mac), T cells, dendritic cells (DC), fibroblasts (Fibro), and smooth muscle cells (SMC) (Figure 7A). Simultaneously, the most prominently upregulated and downregulated genes within each cell cluster were displayed in Figure 7B, along with the proportion of UC samples present in each cell cluster (Figure 7C, 7G). We then categorized the cells in the UC into two vulnerability states based on the median UCRScore, a single peak was observed in the AUC values of all cells, and it was noted that 1027 cells displayed higher score at a threshold of 0.031 (Figure 7D). The percentages of DC, Fibro and SMC increased in the high score, and the percentages of B cell, DC and T cell increased in the low score, suggesting heterogeneity of cellular composition with changing UC status (Figure 7E, 7F). The cell-cell communication network in the UC disease state showed that cell populations under UCRScore-High enhanced the overall weighted afferent/efferent signaling. In particular, Fibro, SMC and Endo showed higher interaction weights under high scores both in terms of the number and weight of intercellular communication (Figure 7H). At high resolution, GSEA further confirmed the differences in disease severity and progression among different UCRScore (Figure 7I). The inflammatory aspects such as focal adhesion and extracellular matrix (ECM) receptor interaction were significantly enriched in the high score, while WNT signal transduction and intestinal immune network were significantly enriched in the low score. We further calculated 2 published biological gene-sets and found that high scores were associated with an increased risk of Dextran Sodium Sulfate (DSS) induced colitis and colorectal polyposis developing and exacerbating UC (Figure 7J).

## DISCUSSION

UC is a common clinical IBD with high morbidity and mortality in west Europe and north America, and the incidence is increasing yearly in developing countries. Untimely and ineffective treatment may induce a series of extraintestinal manifestations such as peripheral arthritis, primary sclerosing cholangitis, and pyoderma gangrenosum [27, 28]. At present, the treatment of ulcerative colitis often begins after the patients have obvious clinical symptoms such as purulent and bloody stool, however, the current clinical routine of painless colonoscopy, ultrasound and fecal calcitonin has no obvious advantages in the early diagnosis of the disease occurrence and often used as a means of diagnosis of UC [7]. A variety of disease activity indexes are established according to the results of clinical, laboratory, and endoscopic examinations, but they are mainly used in clinical trials [29]. The construction of biomarkers with fewer genes means lower cost and easier access to clinical applications [30, 31]. In the time of advocating individualized treatment, it is imperative to determine non-invasive biomarkers to monitor normal people for accurate diagnosis and prevention of UC, so as to optimize the early diagnosis and drug treatment of UC.

This study identified the characteristics of vulnerable biomarkers associated with ulcerative colitis based on differential expression analysis and WGCNA combined with consensus clustering, and provided insights into the potential pathological mechanism and therapeutic targets of the occurrence and progression of UC in terms of bulks and single-cell resolution. Our robust classifier based on UCRGs can effectively distinguish between healthy people and UC patients. The combination of different algorithms can further reduce the number of variables and make the model more concise and transformable.

In this study, we performed a comprehensive evaluation of 8 classical machine learners on their ability to differentiate between normal and UC patients, and combined the best learners to generate a diagnostic model based on UCRGs, which showed good accuracy in predicting clinically relevant outcomes. The combination of different algorithms can further reduce the number of variables, making the model more concise and translational [32]. And the prediction model constructed by us using an integrated learning algorithm showed higher accuracy in both the meta-cohort (AUC=0.977) and the external validation set (the average AUC of the 5 validation cohorts is equal to 0.9588), which shows that the model has extrapolation possible. Meanwhile, compared with the previously reported UC biomarkers that mRNA based on the overexpression of serum neutrophil gelatinase-associated apolipoprotein (NGAL) in the inflammatory intestine, predicting the clinical and endoscopic activity of UC (AUC = 0.758), Fecal Calprotectin (FC) combined with Mayo Endoscopic Score (MES) predicted endoscopic and histological activity of UC patients in clinical remission (AUC = 0.734) and the clinical predictive model based on the ectodomain of type 23 collagen (pro-c23) in serum overexpression in UC patient (AUC = 0.8) [33–35]. The results show that our diagnostic model has higher robustness and extrapolation capability.

However, the occurrence and development of ulcerative colitis is a complex dynamic process, and the detailed mechanism of ulcer formation, development and bleeding is not fully understood. Studies have shown that under prolonged stimulation of inflammation, the barrier function of the intestinal epithelium is damaged, the colonic mucosa induces an autoprotective mechanism, and at the immune synapse between antigen-presenting cells and T lymphocytes, co-stimulation with danger signals is required to induce an effective adaptive immune response [36]. Adaptive immune abnormalities in ulcerative colitis are defined by mucosal CD4+ cells, atypical Th2 responses represented by UC, such as interleukin 13 represented by the presence of secreted atypical natural killer T cells in the colon, intestinal inflammation of the tract is largely dependent on T cell processes, and over the past few decades, the pathogenic mechanisms of T cells have been well defined in research, as well as the characteristics and potential to limit the number of disease-regulating T cells [37, 38].

Traditional models usually only perform macroscopic risk stratification and cannot reflect the actual status of UC patients. At the same time, due to the inherent lack of internal explanation mechanism of machine learning [39], this limits the potential of precision medicine. To address end, we started from an unsupervised perspective and constructed a biologically and clinically interpretable quantification system, called UCRScore, to measure the risk and severity of UC. According to our research results, UC has significant differences in upstream tissue factors or downstream cells and pathways, especially in inflammatory cells such as T cells, neutrophils and plasma cells, as well as IL3, SLC6A14, PECAM1, etc. In terms of tissue factor interaction, in the normal group, important metabolism-related pathway factors such as pre-B cells, bile acid metabolism, mitochondrial fatty acids-oxidized unsaturated fatty acids, and CDX2 were significant enrichment. The GSEA results also showed that UCRGs screened by the integrated classifier also showed high biological activity in terms of intracellular stimulation of biological responses, neutrophil immune activity, inflammatory response, and elastic fiber formation. High UCRScore significantly increases inflammation, stromal, and inflammatory microenvironment scores, which is consistent with inflammatory cells, environmental infiltration, and a series of activated immune-related pathways in the early occurrence of UC [40]. Clinical cytoplasmic proteins secreted by activated neutrophils, including fecal calcium protegerin and fecal lactoferrin, are used as diagnostic biomarkers of IBD, which is consistent with the increased activity of high UCRScore in neutrophils and Th1 cells [41, 42].

UCRScore is a powerful tool that not only characterizes biological states at bulk level but also provides direct snapshots at single-cell resolution. Single-cell RNA sequencing analysis of UC in mouse disease stages showed that high UCRScore occurs in macrophages and fibroblasts both highly expressed. This is due to the long-term inflammatory environment and the coagulation dysfunction of the intestinal mucosa, in the ongoing disease state, the decompensation of intestinal fibroblasts continues to use fibrous connective tissue. Tissue replacement of normal parenchymal tissue leads to irreversible intestinal fibrosis and intestinal stenosis [43]. We found that ECM receptor interactions are enhanced under high UCRScore transition, and metalloproteinases such as matrix metalloproteinases (MMPs) and other proteases activated by chronic inflammation can degrade collagen, elastin, and proteoglycans, leading to ECM structural destruction and sustained ECM remodeling and faulty tissue repair processes may lead to pathological fibrosis, affecting intestinal flexibility and function [44, 45]. In addition, in the UCRScore-High state activates a series of designed tumor necrosis factors, oxidative reactions, and cytokine receptor interactions to aggravate the development of UC. 5-aminosalicylic acid and corticosteroids have been shown to play an important role in the remission and treatment of UC [46, 47]. They inhibit the chemotaxis and activity of leukocytes and reduce the accumulation of inflammatory cells in intestinal mucosa. At the same time, they also inhibit the synthesis of prostaglandins and leukotrienes, which are key mediators in the inflammatory pathway. Immunotherapy is achieved by interfering with the IL-12/ 23axis, JAK and TGF- β / Smad7 pathways, and regulating IL-6, chemokine and chemokine receptors CC receptor 9-CC chemokine ligand 25 (CCR9-CCL25), cell adhesion and leukocyte recruitment [48]. At present, a variety of biological agents have been approved for the immunotherapy of IBD, including infliximab, vedolizumab and natalizumab. Computational analysis has been popularized and widely used in pharmacological development and testing, and it is often used to discover and optimize new molecules with affinity to the target [49]. Therefore, based on CMap analysis and the principle of UC occurrence, we identified five potential targeted drugs that are most likely to reverse the expression level of UCRGs, they may play a potential role in cell membrane stability, anti-inflammatory effect and mucosal protection to intervene the occurrence of UC, including clofibrate, fasudil, MK.866, NU.1025 and imatinib. It can be used as an additional supplement to the therapeutic of UC.

## CONCLUSIONS

In conclusion, this study uses computational biology methods to comprehensively analyze vulnerability to ulcerative colitis, which will provide broad biological and clinical perspectives for future functional and therapeutic studies on the occurrence and progression of UC. The high-dimensional quantification system UCRScore has a powerful ability to measure the occurrence and severity of UC, and may serve as a tool to optimize clinical decision-making and management of UC patients.

## Supplementary Material

Supplementary Figures

Supplementary Tables 1 and 2

Supplementary Table 3

Supplementary Table 4
