# Supplementary material for: An assessment system for clinical and biological interpretability in ulcerative colitis
Source: Aging (Albany NY). 2024 Feb 16;16(4):3856–79. doi: 10.18632/aging.205564 (PMC10929837; doi:10.18632/aging.205564)
Supplement: Supplementary Figures [file aging-16-205564-s001.pdf]

SUPPLEMENTARY FIGURES

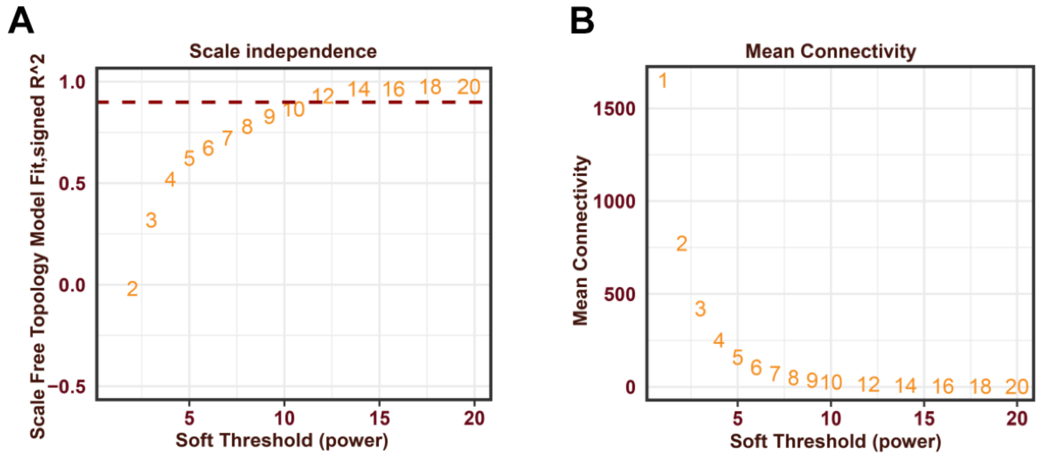

Supplementary Figure 1. Determine the optimal soft threshold for WGCNA independence index and average connectivity. (**A**) Estimation of the scale independence index of the 1–20 soft threshold power (β = 14). (**B**) Determination of the mean connectivity of the 1–20 soft threshold power.

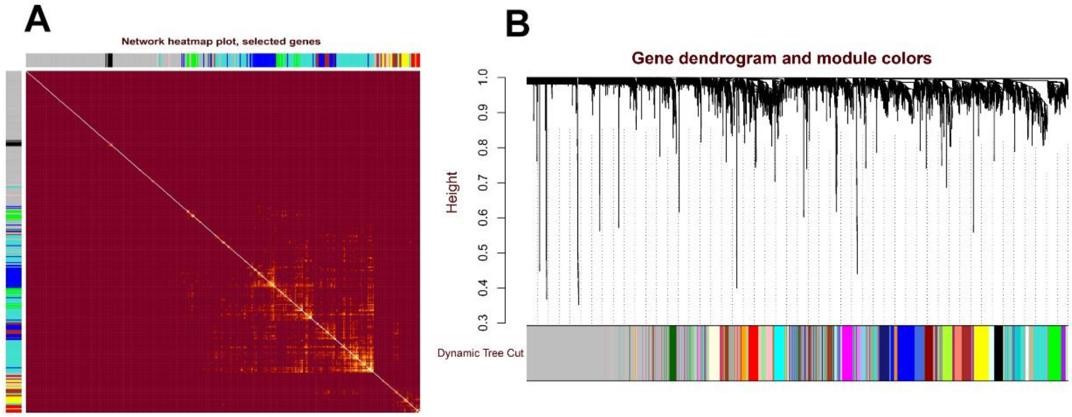

Supplementary Figure 2. Weighted gene co-expression analysis (WGCNA) based on the common DEGs. (**A**) Heatmap depicting the topological overlap matrix among all genes in WGCNA. (**B**) Gene clustering diagram based on hierarchical clustering under optimal soft-thresholding power as 14. Genes with similar expression patterns were assigned to co-expression modules represented by the same color.

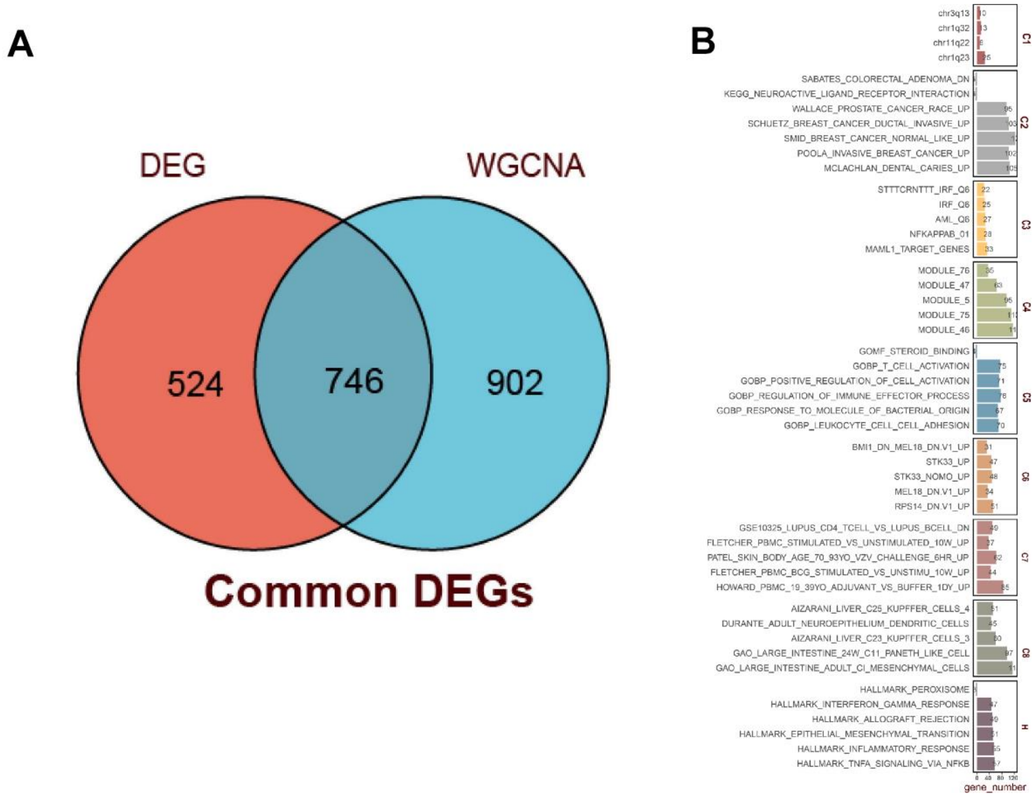

Supplementary Figure 3. Uncovering dysregulated gene co-expression pattern and ORA analysis. (**A**) Venn diagram showing co-expressed genes in DEGs and WGCNA modules. (**B**) The barplot shows up-and-down-regulated genes in common DEGs by ORA based on the C1-C8 and Hallmark datasets obtained from MsigDB database.

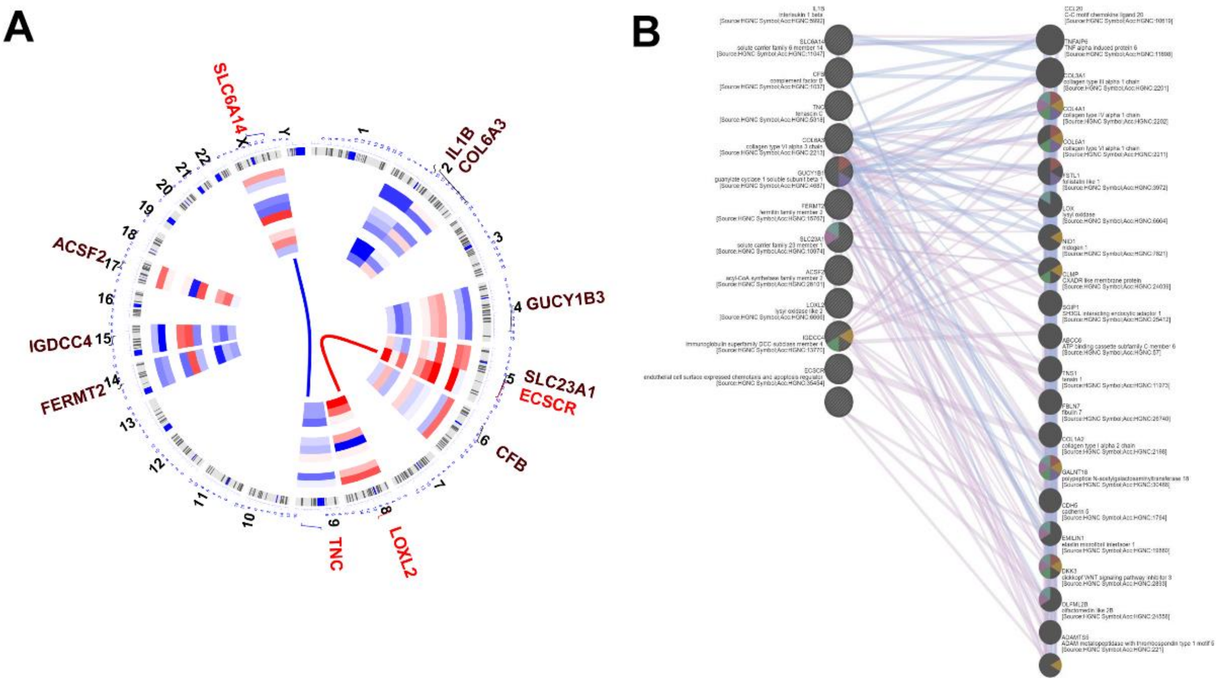

Supplementary Figure 4. Chromosome location and genetic interactions of UCRGs. (**A**) Identify the chromosome location of UCRGs. (**B**) Genetic interaction and co-expression patterns about UCRGs.

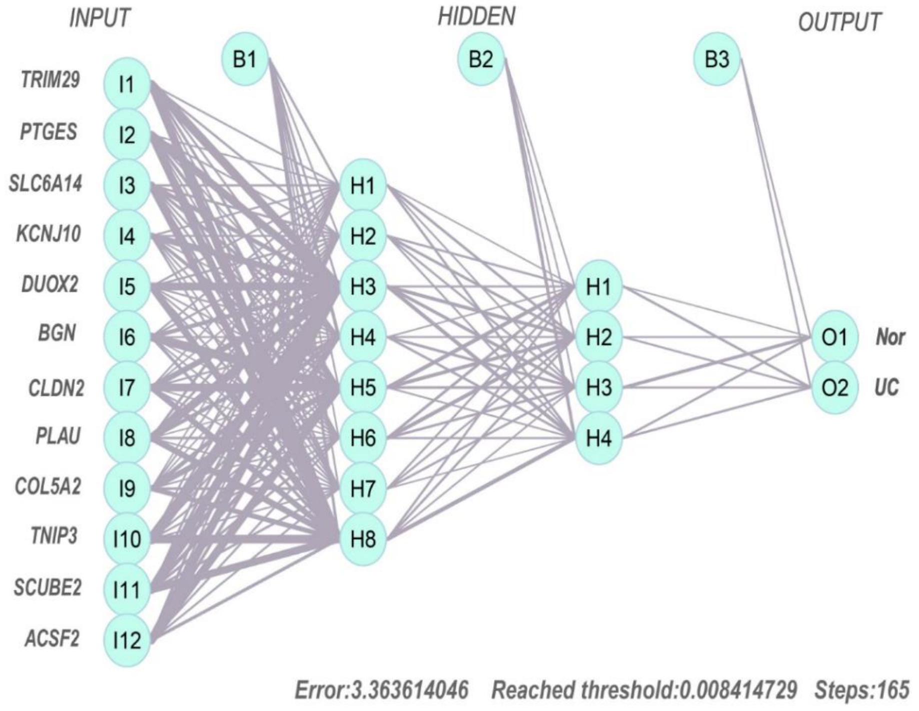

Supplementary Figure 5. Results of neural network visualization. Visualization of UCRGs classifier: A neural network was trained from UCRGs using backpropagation for patient diagnosis.

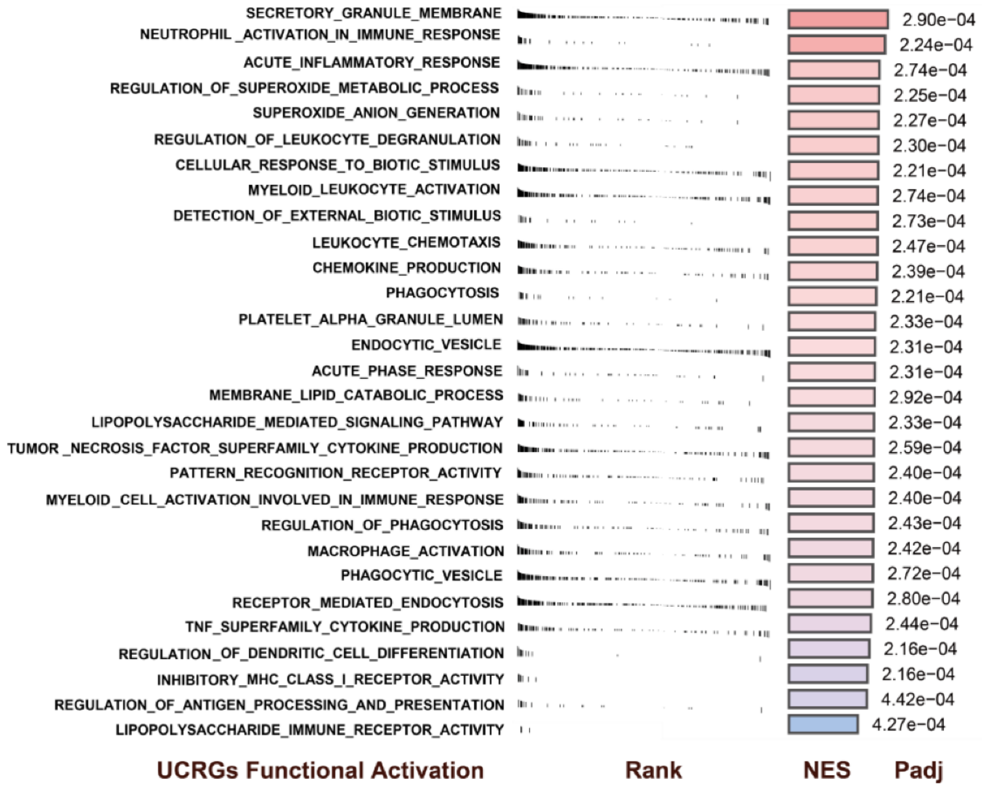

Supplementary Figure 6. GSEA analysis of biological process pathway of UCRGs. GSEA estimates differences in biological pathway activity of UCRGs.
