# Supplementary material for: An assessment system for clinical and biological interpretability in ulcerative colitis
Source: Aging (Albany NY). 2024 Feb 16;16(4):3856–79. doi: 10.18632/aging.205564 (PMC10929837; doi:10.18632/aging.205564)
Supplement: Supplementary Tables 1 and 2 [file aging-16-205564-s002.pdf]

SUPPLEMENTARY TABLES

Supplementary Table 1. Details of public datasets used in this study.

No	Dataset	Source	Sample type	Organism	Number and description of samples in this study	Sample size	PubMed ID
1	GSE87466	Li K, et al.	Mucosal biopsy samples	Homo sapiens	21 Healthy controls, 87 UC patients	108	PMID:29401083
2	GSE47908	Bjerrum JT et al.	Colonic biopsies	Homo sapiens	19 Healthy controls, 41 UC patients	60	PMID: 25358065
3	GSE59071	Arijs I et al.	Mucosal biopsies	Homo sapiens	16 Healthy controls, 92 UC patients	108	PMID: 26313692
4	GSE75214	Arijs I et al.	Mucosal biopsies	Homo sapiens	19 Healthy controls, 100 UC patients	119	PMID: 28885228
5	GSE92415	Li Ket al.	Mucosal biopsy samples	Homo sapiens	20 Healthy controls, 54 UC patients	74	PMID: 23735746
6	GSE14580	Arijs I et al.	Mucosal biopsy samples	Homo sapiens	6 Healthy controls, 24 UC patients	30	PMID: 19700435
7	GSE53306	Zhao X et al.	Mucosal biopsies	Homo sapiens	16 Healthy controls, 12 Inactive UC, 12 Active UC	40	PMID: 26034135
8	GSE13367	Bjerrum JT et al.	mucosal colonic biopsies	Homo sapiens	16 Infiltrative UC, 18 Non-infiltrative UC	34	PMID: 19834973
9	GSE16879	Arijs I et al.	Mucosal biopsies	Homo sapiens	8 Infliximab Responsive UC, 16 Infliximab Irresponsive UC	24	PMID: 19956723
10	GSE6731	Wu F et al.	Mucosal biopsies	Homo sapiens	5 Infiltrative UC, 4 Non-infiltrative UC	9	PMID: 17262512
**Total sample size** 606							

Supplementary Table 2. A total of 9 UC occurrence relative genes are retrieved from the literature.

Gene	PMID	Author	ENSEMBL
**ZC3H12A**	33359885	Kyle Gettler	ENSG00000163874
**STMN3**	21297633	Carl A Anderson	ENSG00000197457
**IL7R**	21297633	Carl A Anderson	ENSG00000168685
**RTEL1**	21297633	Carl A Anderson	ENSG00000258366
**NOD2**	26490195	Isabelle Cleynen	ENSG00000167207
**JAK2**	31853061	Nobuyuki Kakiuchi	ENSG00000096968
**SLC22A5**	16361305	S Waller	ENSG000000197375
**CFB**	24837172	Garima Juyal	ENSG000000243649
**TLR2**	16480927	Elisabet Cantó	ENSG000000137462
