# Supplementary material for: An assessment system for clinical and biological interpretability in ulcerative colitis
Source: Aging (Albany NY). 2024 Feb 16;16(4):3856–79. doi: 10.18632/aging.205564 (PMC10929837; doi:10.18632/aging.205564)
Supplement: Supplementary Table 3 [file aging-16-205564-s003.docx]

**Supplementary Table 3.** **The detailed information on feature weight in BP neural network.**

| error | 3.361030098 |
| --- | --- |
| reached.threshold | 0.008771159 |
| steps | 175 |
| Intercept.to.1layhid1 | -0.753757261 |
| SLC6A14.to.1layhid1 | -0.269628159 |
| CFB.to.1layhid1 | -0.155680191 |
| ECSCR.to.1layhid1 | -1.533456304 |
| COL6A3.to.1layhid1 | -0.319008346 |
| IL1B.to.1layhid1 | -0.623629155 |
| FERMT2.to.1layhid1 | -1.710578065 |
| GUCY1B3.to.1layhid1 | 0.078086981 |
| TNC.to.1layhid1 | -1.778979221 |
| IGDCC4.to.1layhid1 | -0.983668919 |
| LOXL2.to.1layhid1 | -2.382913422 |
| ACSF2.to.1layhid1 | -1.873069083 |
| SLC23A1.to.1layhid1 | -2.617691653 |
| Intercept.to.1layhid2 | 1.262815763 |
| SLC6A14.to.1layhid2 | 0.932777202 |
| CFB.to.1layhid2 | 2.517658293 |
| ECSCR.to.1layhid2 | 0.27770523 |
| COL6A3.to.1layhid2 | -1.574650136 |
| IL1B.to.1layhid2 | 0.593772191 |
| FERMT2.to.1layhid2 | 3.693925536 |
| GUCY1B3.to.1layhid2 | 0.473142682 |
| TNC.to.1layhid2 | 1.282575184 |
| IGDCC4.to.1layhid2 | 1.567792869 |
| LOXL2.to.1layhid2 | -0.76856055 |
| ACSF2.to.1layhid2 | -0.007668007 |
| SLC23A1.to.1layhid2 | 2.828978879 |
| Intercept.to.1layhid3 | 1.062483949 |
| SLC6A14.to.1layhid3 | -17.62506654 |
| CFB.to.1layhid3 | -12.74639151 |
| ECSCR.to.1layhid3 | -15.35166844 |
| COL6A3.to.1layhid3 | -7.531778945 |
| IL1B.to.1layhid3 | -15.46229749 |
| FERMT2.to.1layhid3 | -9.145265181 |
| GUCY1B3.to.1layhid3 | -3.534206891 |
| TNC.to.1layhid3 | -2.133418932 |
| IGDCC4.to.1layhid3 | -13.53311782 |
| LOXL2.to.1layhid3 | -19.46567085 |
| ACSF2.to.1layhid3 | -15.92092191 |
| SLC23A1.to.1layhid3 | -12.98248418 |
| Intercept.to.1layhid4 | 0.251309676 |
| SLC6A14.to.1layhid4 | -0.967866492 |
| CFB.to.1layhid4 | -8.835682473 |
| ECSCR.to.1layhid4 | -13.7399085 |
| COL6A3.to.1layhid4 | -8.216534917 |
| IL1B.to.1layhid4 | 0.324710249 |
| FERMT2.to.1layhid4 | -2.754513852 |
| GUCY1B3.to.1layhid4 | -4.032426853 |
| TNC.to.1layhid4 | 0.480455982 |
| IGDCC4.to.1layhid4 | -15.44546981 |
| LOXL2.to.1layhid4 | -12.47854206 |
| ACSF2.to.1layhid4 | -14.70603359 |
| SLC23A1.to.1layhid4 | -11.89424923 |
| Intercept.to.1layhid5 | -0.671071134 |
| SLC6A14.to.1layhid5 | 0.43493365 |
| CFB.to.1layhid5 | -1.141479249 |
| ECSCR.to.1layhid5 | 0.787059755 |
| COL6A3.to.1layhid5 | 0.802435728 |
| IL1B.to.1layhid5 | -0.472916979 |
| FERMT2.to.1layhid5 | 1.147975291 |
| GUCY1B3.to.1layhid5 | -0.403567863 |
| TNC.to.1layhid5 | 1.143904096 |
| IGDCC4.to.1layhid5 | 0.15776375 |
| LOXL2.to.1layhid5 | 0.393048843 |
| ACSF2.to.1layhid5 | 0.936772862 |
| SLC23A1.to.1layhid5 | 0.610879006 |
| Intercept.to.1layhid6 | 0.578487181 |
| SLC6A14.to.1layhid6 | 0.162707199 |
| CFB.to.1layhid6 | -0.90528609 |
| ECSCR.to.1layhid6 | 0.472166655 |
| COL6A3.to.1layhid6 | 0.812711034 |
| IL1B.to.1layhid6 | -0.098890024 |
| FERMT2.to.1layhid6 | 0.86611577 |
| GUCY1B3.to.1layhid6 | 0.018820049 |
| TNC.to.1layhid6 | 0.672563953 |
| IGDCC4.to.1layhid6 | -0.304242498 |
| LOXL2.to.1layhid6 | 0.332072033 |
| ACSF2.to.1layhid6 | 0.885996027 |
| SLC23A1.to.1layhid6 | 0.358413469 |
| Intercept.to.1layhid7 | -0.45977605 |
| SLC6A14.to.1layhid7 | -0.985285688 |
| CFB.to.1layhid7 | 0.057811627 |
| ECSCR.to.1layhid7 | -0.842981178 |
| COL6A3.to.1layhid7 | -0.303410856 |
| IL1B.to.1layhid7 | -0.295016679 |
| FERMT2.to.1layhid7 | -0.314688387 |
| GUCY1B3.to.1layhid7 | -0.205380181 |
| TNC.to.1layhid7 | -0.710237449 |
| IGDCC4.to.1layhid7 | 0.126890555 |
| LOXL2.to.1layhid7 | 0.295635769 |
| ACSF2.to.1layhid7 | -1.076251283 |
| SLC23A1.to.1layhid7 | -0.982641159 |
| Intercept.to.1layhid8 | 0.660144743 |
| SLC6A14.to.1layhid8 | 1.337884846 |
| CFB.to.1layhid8 | 3.04965863 |
| ECSCR.to.1layhid8 | 1.154442748 |
| COL6A3.to.1layhid8 | -2.496468969 |
| IL1B.to.1layhid8 | 0.209544662 |
| FERMT2.to.1layhid8 | 0.909545058 |
| GUCY1B3.to.1layhid8 | 1.097416691 |
| TNC.to.1layhid8 | -0.469962251 |
| IGDCC4.to.1layhid8 | 1.29513244 |
| LOXL2.to.1layhid8 | 0.893196481 |
| ACSF2.to.1layhid8 | 0.295433311 |
| SLC23A1.to.1layhid8 | 3.501763325 |
| Intercept.to.1layhid9 | -0.487880904 |
| SLC6A14.to.1layhid9 | -16.10619167 |
| CFB.to.1layhid9 | -15.21899117 |
| ECSCR.to.1layhid9 | -14.67603002 |
| COL6A3.to.1layhid9 | -7.415350683 |
| IL1B.to.1layhid9 | -16.28792654 |
| FERMT2.to.1layhid9 | -9.016200546 |
| GUCY1B3.to.1layhid9 | -2.847164132 |
| TNC.to.1layhid9 | -2.773204749 |
| IGDCC4.to.1layhid9 | -15.52323278 |
| LOXL2.to.1layhid9 | -15.90560833 |
| ACSF2.to.1layhid9 | -16.83629719 |
| SLC23A1.to.1layhid9 | -3.412476542 |
| Intercept.to.1layhid10 | -0.658420196 |
| SLC6A14.to.1layhid10 | 1.493904461 |
| CFB.to.1layhid10 | 1.471816664 |
| ECSCR.to.1layhid10 | 1.277329732 |
| COL6A3.to.1layhid10 | -1.286684447 |
| IL1B.to.1layhid10 | -0.18789811 |
| FERMT2.to.1layhid10 | 0.89298999 |
| GUCY1B3.to.1layhid10 | 0.242390793 |
| TNC.to.1layhid10 | -0.093532883 |
| IGDCC4.to.1layhid10 | 1.942644052 |
| LOXL2.to.1layhid10 | 0.835024829 |
| ACSF2.to.1layhid10 | -0.009461703 |
| SLC23A1.to.1layhid10 | 2.936831069 |
| Intercept.to.2layhid1 | -0.485037659 |
| 1layhid1.to.2layhid1 | -3.740136208 |
| 1layhid2.to.2layhid1 | -0.460864761 |
| 1layhid3.to.2layhid1 | -1.457946338 |
| 1layhid4.to.2layhid1 | 0.447572157 |
| 1layhid5.to.2layhid1 | -2.218624188 |
| 1layhid6.to.2layhid1 | -0.30269058 |
| 1layhid7.to.2layhid1 | -2.517937348 |
| 1layhid8.to.2layhid1 | 0.280547368 |
| 1layhid9.to.2layhid1 | -1.387288108 |
| 1layhid10.to.2layhid1 | -1.109929758 |
| Intercept.to.2layhid2 | -0.267979748 |
| 1layhid1.to.2layhid2 | 0.830425901 |
| 1layhid2.to.2layhid2 | -0.417789735 |
| 1layhid3.to.2layhid2 | 0.537915376 |
| 1layhid4.to.2layhid2 | 0.880389561 |
| 1layhid5.to.2layhid2 | -0.059957294 |
| 1layhid6.to.2layhid2 | 0.220006439 |
| 1layhid7.to.2layhid2 | 0.450853418 |
| 1layhid8.to.2layhid2 | 0.044093569 |
| 1layhid9.to.2layhid2 | -0.097607205 |
| 1layhid10.to.2layhid2 | 0.955185863 |
| Intercept.to.2layhid3 | -1.069637785 |
| 1layhid1.to.2layhid3 | 0.200687065 |
| 1layhid2.to.2layhid3 | -0.687560563 |
| 1layhid3.to.2layhid3 | 0.27815232 |
| 1layhid4.to.2layhid3 | 0.477195558 |
| 1layhid5.to.2layhid3 | 1.385677716 |
| 1layhid6.to.2layhid3 | -0.94908017 |
| 1layhid7.to.2layhid3 | 1.325029865 |
| 1layhid8.to.2layhid3 | 2.681277363 |
| 1layhid9.to.2layhid3 | 0.209567159 |
| 1layhid10.to.2layhid3 | -0.243151354 |
| Intercept.to.2layhid4 | 0.504288526 |
| 1layhid1.to.2layhid4 | 0.386693419 |
| 1layhid2.to.2layhid4 | 1.580611878 |
| 1layhid3.to.2layhid4 | -0.853736109 |
| 1layhid4.to.2layhid4 | 0.228053705 |
| 1layhid5.to.2layhid4 | -1.293038827 |
| 1layhid6.to.2layhid4 | 0.407737772 |
| 1layhid7.to.2layhid4 | 1.07385778 |
| 1layhid8.to.2layhid4 | 1.097268603 |
| 1layhid9.to.2layhid4 | 1.592757717 |
| 1layhid10.to.2layhid4 | -0.247356772 |
| Intercept.to.2layhid5 | -0.462611235 |
| 1layhid1.to.2layhid5 | -0.094467537 |
| 1layhid2.to.2layhid5 | 1.339542559 |
| 1layhid3.to.2layhid5 | -1.324231075 |
| 1layhid4.to.2layhid5 | 0.445099312 |
| 1layhid5.to.2layhid5 | 0.749093144 |
| 1layhid6.to.2layhid5 | -2.00165488 |
| 1layhid7.to.2layhid5 | 0.39817855 |
| 1layhid8.to.2layhid5 | 1.370639986 |
| 1layhid9.to.2layhid5 | -1.807301445 |
| 1layhid10.to.2layhid5 | 1.120135507 |
| Intercept.to.2layhid6 | -0.800330113 |
| 1layhid1.to.2layhid6 | 0.587338674 |
| 1layhid2.to.2layhid6 | 1.309186315 |
| 1layhid3.to.2layhid6 | 0.859127404 |
| 1layhid4.to.2layhid6 | 0.24561172 |
| 1layhid5.to.2layhid6 | -0.140756652 |
| 1layhid6.to.2layhid6 | -1.031039414 |
| 1layhid7.to.2layhid6 | -1.552476454 |
| 1layhid8.to.2layhid6 | -0.12496695 |
| 1layhid9.to.2layhid6 | 0.402692634 |
| 1layhid10.to.2layhid6 | -1.508043577 |
| Intercept.to.Nor | -0.650913025 |
| 2layhid1.to.Nor | 1.689163499 |
| 2layhid2.to.Nor | 1.432066061 |
| 2layhid3.to.Nor | 0.779558023 |
| 2layhid4.to.Nor | -0.177194786 |
| 2layhid5.to.Nor | -0.848760124 |
| 2layhid6.to.Nor | 0.839899547 |
| Intercept.to.UC | 0.666604833 |
| 2layhid1.to.UC | 0.359126194 |
| 2layhid2.to.UC | -1.989681804 |
| 2layhid3.to.UC | 0.629371123 |
| 2layhid4.to.UC | 0.316097788 |
| 2layhid5.to.UC | 0.924981081 |
| 2layhid6.to.UC | -0.309319699 |
